# Supplementary material for: Climate Change Influences the Population Density and Suitable Area of Hippotiscus dorsalis (Hemiptera: Pentatomidae) in China
Source: Insects. 2023 Jan 28;14(2):135. doi: 10.3390/insects14020135 (PMC9963971; doi:10.3390/insects14020135)
Supplement: Supplementary file 1 [file insects-14-00135-s001.zip › insects-1946401-supplementary.pdf]

Supplementary Materials: The following are available online at https://www.mdpi.com/article/10.3390/insects14020135/s1, Figure S1: Map of Chinese provinces. Table S1: Occurrence records coord of *Hippotiscus dorsalis* in China.

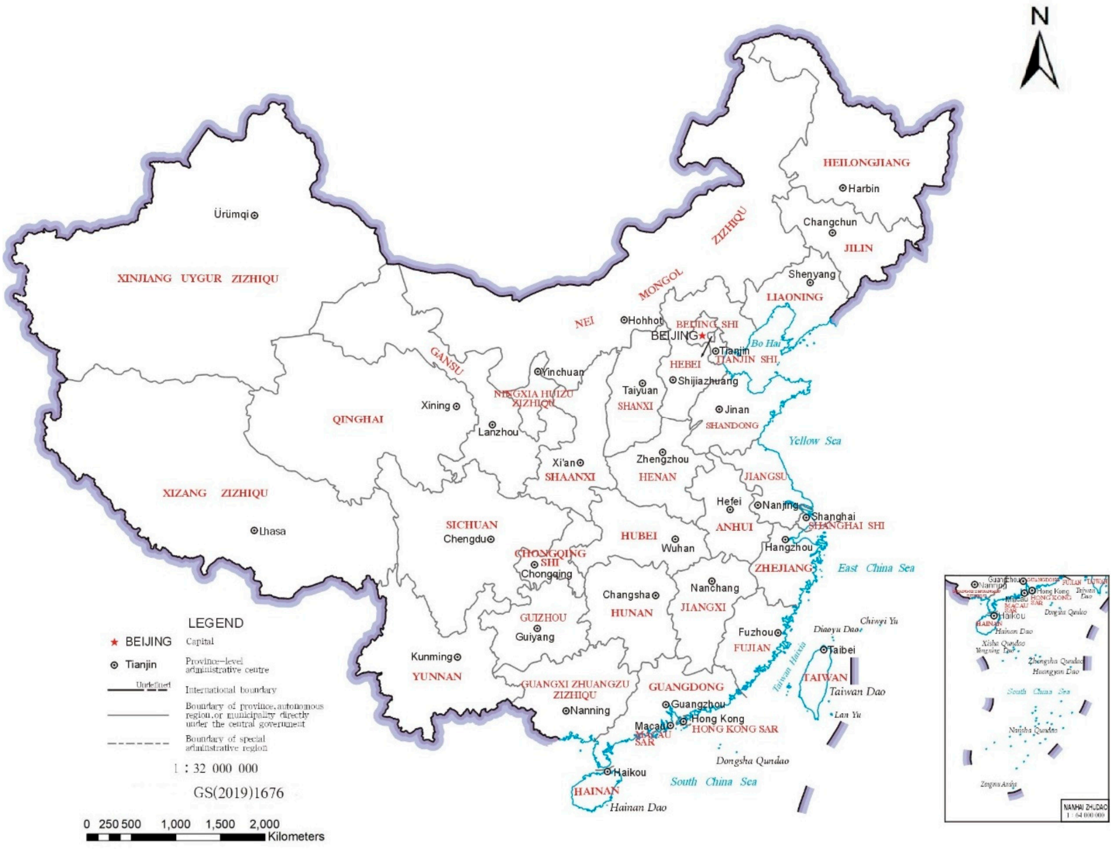

Figure S1. Map of Chinese provinces.

Table S1. Occurrence records coord of *Hippotiscus dorsalis* in China.

species	longitude	latitude
Hippota dorsalis	121.7168	29.9545
Hippota dorsalis	121.6887	29.4874
Hippota dorsalis	119.5202	29.0858
Hippota dorsalis	120.4814	29.991
Hippota dorsalis	119.0989	28.4823
Hippota dorsalis	120.0322	28.4692
Hippota dorsalis	120.3456	30.4185
Hippota dorsalis	118.5844	30.7062
Hippota dorsalis	119.0873	30.6341
Hippota dorsalis	116.917	27.7576
Hippota dorsalis	115.8591	28.176
Hippota dorsalis	115.4867	27.7335
Hippota dorsalis	118.0363	28.4574

Hippota dorsalis	118.2981	28.689
Hippota dorsalis	116.2014	27.7526
Hippota dorsalis	111.6856	29.0286
Hippota dorsalis	113.6494	26.5051
Hippota dorsalis	105.7121	28.4547
Hippota dorsalis	105.9544	28.3769
Hippota dorsalis	121.3583	29.41653
Hippota dorsalis	121.3679	29.29622
Hippota dorsalis	121.8554	29.47083
Hippota dorsalis	121.2788	29.60117
Hippota dorsalis	121.3798	29.94599
Hippota dorsalis	121.1221	29.74003
Hippota dorsalis	121.5534	29.81811
Hippota dorsalis	121.336	29.50568
Hippota dorsalis	121.217	29.87284
Hippota dorsalis	121.2218	29.46282
Hippota dorsalis	121.4127	29.66414
Hippota dorsalis	121.4923	29.91366
Hippota dorsalis	121.1586	30.05224
Hippota dorsalis	121.6275	29.3665
Hippota dorsalis	118.8651	28.97
Hippota dorsalis	118.729	29.07885
Hippota dorsalis	118.8269	28.76954
Hippota dorsalis	119.3137	29.03877
Hippota dorsalis	119.1705	28.99101
Hippota dorsalis	119.2094	28.90957
Hippota dorsalis	119.088	28.8848
Hippota dorsalis	119.3956	30.58224
Hippota dorsalis	119.5614	30.78502
Hippota dorsalis	119.981	30.54071
Hippota dorsalis	119.8932	30.74478
Hippota dorsalis	119.5111	30.56344
Hippota dorsalis	120.1127	30.87941
Hippota dorsalis	119.8757	30.64795
Hippota dorsalis	119.9171	31.03388
Hippota dorsalis	119.7391	30.56299
Hippota dorsalis	119.882	30.48443
Hippota dorsalis	120.0536	30.38597
Hippota dorsalis	119.9528	30.27729
Hippota dorsalis	119.8971	30.20266
Hippota dorsalis	120.2445	30.19775
Hippota dorsalis	119.036	29.59812
Hippota dorsalis	119.1014	29.47402
Hippota dorsalis	119.5615	29.85397

Hippota dorsalis	120.0342	30.00358
Hippota dorsalis	120.0482	30.12322
Hippota dorsalis	119.0091	27.6582
Hippota dorsalis	119.7581	28.30193
Hippota dorsalis	119.1762	28.58989
Hippota dorsalis	119.1666	28.70257
Hippota dorsalis	118.9582	28.4627
Hippota dorsalis	119.2568	28.64259
Hippota dorsalis	119.4769	28.44617
Hippota dorsalis	119.9106	28.51188
Hippota dorsalis	118.9571	27.96878
Hippota dorsalis	119.299	28.54539
Hippota dorsalis	119.0231	28.70916
Hippota dorsalis	119.3188	28.7288
Hippota dorsalis	119.4316	28.87068
Hippota dorsalis	119.6633	28.96913
Hippota dorsalis	119.8271	28.75227
Hippota dorsalis	119.4365	30.35379
Hippota dorsalis	120.5802	30.00838
Hippota dorsalis	120.9171	30.00572
Hippota dorsalis	114.3384	25.67779
Hippota dorsalis	117.0614	27.67756
Hippota dorsalis	105.9461	28.48726
Hippota dorsalis	118.5554	30.28026
Hippota dorsalis	118.6099	30.06229
Hippota dorsalis	118.3917	30.76032
Hippota dorsalis	118.1569	30.48639
Hippota dorsalis	118.9924	30.89798
Hippota dorsalis	118.78	30.76647
Hippota dorsalis	118.8875	30.91084
Hippota dorsalis	118.9814	30.79597
Hippota dorsalis	118.6961	30.69427
Hippota dorsalis	118.738	30.94912
Hippota dorsalis	109.7964	27.2215
Hippota dorsalis	111.6447	27.7258
Hippota dorsalis	110.8408	26.42808
Hippota dorsalis	113.7306	25.57718
Hippota dorsalis	112.6442	28.22619
Hippota dorsalis	111.5675	28.71053
Hippota dorsalis	110.6427	26.69934
